# Comparing the health of refugee and asylee patients with that of non-refugee immigrant and US-born patients in a large Urban clinic

**DOI:** 10.1186/s12889-023-16349-5

**Published:** 2023-07-27

**Authors:** Eva Raphael, Michelle Barton, Katrin Jaradeh, Cristy Dieterich, Rita Hamad

**Affiliations:** 1 Department of Epidemiology & Biostatistics, University of California San Francisco (UCSF), San Francisco, CA USA; 2 Department of Family & Community Medicine, UCSF, San Francisco, CA USA; 3 Department of Behavioral Health, Bozeman Health Deaconess Hospital, Bozeman, MT USA; 4 Department of Emergency Medicine, UCSF, San Francisco, CA USA; 5 Newcomers Health Program: SF Refugee Health Assessment Program Community Health Equity & Promotion Branch, Department of Public Health, San Francisco, SF USA; 6 Philip R. Lee Institute for Health Policy Studies, UCSF, San Francisco, CA USA

**Keywords:** Refugee, Asylee, Disease burden, Epidemiology, Immigrants

## Abstract

**Objectives:**

To compare disease burden in refugee/asylee, non-refugee immigrant, and US-born patients in the largest safety net clinic in San Francisco, California.

**Methods:**

This is a retrospective chart review including 343 refugee/asylee, 450 immigrant, and 202 US-born patients in a San Francisco clinic from January 2014 to December 2017. Using electronic medical records, we compared prevalence of several diseases by immigration status. Using Poisson regression models with robust variance, we assessed association of diseases with immigration status, adjusting for sociodemographic characteristics.

**Results:**

Diagnoses of non-communicable chronic diseases were less common in refugees/asylees, who had a greater risk of being diagnosed with mental health conditions. In Poisson regression models adjusted for sociodemographic characteristics, compared with refugees/asylees, US-born patients were more likely to have hypertension (IRR[CI] = 1.8 [1.0, 3.7]) and less likely to have depression (IRR[CI] = 0.5 [0.3, 0.8]). US-born (IRR[CI] = 0.06 [0.01, 0.2]) and immigrant patients (IRR[CI] = 0.1 [0.06, 0.2]) were less likely to have post-traumatic stress disorder.

**Conclusions:**

We uncover differences in burden of non-communicable chronic diseases and mental health by immigration status. These results highlight the importance of clinical screenings and research on disease burden in refugees.

**Supplementary Information:**

The online version contains supplementary material available at 10.1186/s12889-023-16349-5.

## Background

With each geopolitical crisis come waves of forcibly displaced people [1]. While international media and responding political states focus on new crises, there is little research on the long-term outcomes of refugees and asylees. Refugees and asylum seekers are people who have had to leave their home country and are unable to return due to fear of persecution, conflict, violence, or human rights violations [2]. Refugees are accepted for admission into a new country prior to leaving their home country and asylum seekers apply for asylum after arrival to a new country. Asylees have been granted asylum status. Both groups are socially and economically marginalized, which puts them at increased risk for worse long-term mental and physical health [3, 4]. Drivers of health inequities by immigration status include unequal access to and utilization of healthcare services, differential health behaviors, and social determinants of health specific to immigration, including the experience of xenophobia [5, 6]. Most studies focus on the immediate period after resettlement [7–9]. For example, in 2016, a wealth of knowledge came from studies in Europe, emphasizing the immediate medical needs of Syrian refugees [10, 11]. While readily available databases on refugee populations or immigrant communities exist in the US, they are often constrained to data from refugee health assessment clinics or cross-sectional self-reported data from the New Immigrant Survey [3, 12, 13].

The US received more than 438,000 refugees between 2014 to 2017 [14]. In 2022, only 25,465 refugees were granted status, representing half of the historic yearly average in the US [15]. A quarter of all US refugees are accepted in the states of California, New York, and Texas [16]. In California, eleven counties host refugee health assessment clinics that offer comprehensive health screens within the first 90 days of arrival, referrals to primary care clinics, and mental health and legal services [13, 17]. There has been recent interest in better understanding both the prevalence of non-communicable chronic diseases, such as diabetes or cardiovascular disease, that refugees may experience and the unique challenges they may face in accessing appropriate clinical care [18]. A scoping review reported that refugees resettled in the US had higher odds of having a chronic health condition compared to non-refugee immigrants [19]. A study in Germany showed similar findings when asylum seekers were compared to German residents [20]. Yet, to date, few studies have thoroughly characterized the prevalence of non-communicable chronic diseases in refugee populations in the US, and most existing studies have focused on groups from specific countries or on specific diseases [19, 21, 22]. Moreover, more information is needed about how the health of refugees and asylees, specifically, compares to other immigrants in the US as access to healthcare differs by immigration status [23]. This is in part due to limited follow-up, as most studies report on mental health and communicable diseases from initial health screens upon arrival to the US [8, 10, 24].

Here, we seek to fill this gap by comparing the burden of communicable and non-communicable chronic diseases, as well as mental health conditions, among refugees and asylees compared to non-refugee immigrants and US-born Americans receiving care in the same large urban safety net clinic. We hypothesize that refugee/asylee patients have a higher disease burden compared to non-refugee immigrant and US-born patients given their socioeconomic vulnerability and decreased access to health care in their home countries. This work contributes critically needed evidence on an understudied population to better inform the allocation of resources and planning for specific health interventions.

## Methods

### Study site

The Newcomers Health Program (NHP), a program of the San Francisco Department of Public Health, oversees care coordination and Refugee Health Assessments for recently arrived refugees and asylees recently granted asylum status in three San Francisco Bay Area counties. The NHP conducts multiple activities related to immigration and access to healthcare. The NHP coordinates outreach to potential asylum seekers in the community. NHP staff, most of whom are health workers and immigrants themselves, provide health coaching, connections to community-based organizations to help with employment and educational opportunities amongst other resources, and support with changing immigration status (i.e., applying for a green card). Importantly, the NHP coordinates Refugee Health Assessments which are mandatory for both refugees newly arrived in the US and asylees (who have received asylum status). The NHP is located in the Family Health Center, the largest primary care clinic within the San Francisco Health Network, a public safety net healthcare system. The Family Health Center serves about 12,000 patients per year, [25] of which about 200 access the NHP. Approximately a third of all patients within the clinic speak languages other than English as their primary language and interpretation services are made available for all visit types [26] The Refugee Health Assessments are conducted by trained clinicians over 2 visits, using a standard format. Patients are asked about sociodemographic characteristics and type of persecution experienced and screened for communicable and non-communicable diseases, as well as mental health disorders (Supplemental Table 1). The examinations are carried out in the patient’s preferred language, with the use of in-person interpreters when available or phone interpretation services. Data from the Refugee Health Assessment is entered both in the electronic medical records (EMR) system and in RHEIS, a web-based database developed in 2013 to transmit, standardize, and generate reports on refugee health screening data used by the California Department of Public Health.

### Study design

This is a retrospective chart review of adult patients 18 years and older receiving care at the Family Health Center. All refugee and asylee patients receiving NHP services from 2014 to 2017 were included in the study, as identified from RHEIS. Refugees and asylees who resided in cities other than San Francisco or who received primary care services in clinics other than the Family Health Center were excluded. A random unmatched sample of non-refugee/asylee patients identified from a list of all patients at the Family Health Center, who were either non-refugee/asylee immigrants (N = 450) or US-born (N = 202), was also selected. All power calculations assume 80% power (1 – beta) and a 2-sided alpha of 0.05. In this study, we tested whether immigration status (refugee/asylee, immigrant, and US-born) was associated with having various medical conditions. To detect a relative risk of 2.00 of having diabetes, for example, in refugee/asylee patients compared to US-born patients, a sample size of 196 refugee/asylee patients and 196 US-born patients would be needed.

### Data collection

We obtained data from RHEIS from NHP and Family Health Center databases from patients having clinic visits from January 2014 to December 2017. Through manual chart abstraction, trained data collectors obtained sociodemographic information and relevant dates from structured EMR data fields and unstructured clinical notes. All medical conditions of interest were collected from problem lists containing International Classification of Diseases (ICD) codes. At least 5 primary care visit notes, including the first visit when available in the EMR as some patients had started receiving care when paper charts were still in use, were read in their entirety to determine the presence of these data. Data were entered and managed using a REDCap (Research Electronic Data Capture) electronic data capture tool hosted at the first author’s institution [27, 28]. REDCap is a secure, web-based software platform designed to support data capture for research studies, providing (1) an intuitive interface for validated data capture; (2) audit trails for tracking data manipulation and export procedures; (3) automated export procedures for seamless data downloads to common statistical packages; and (4) procedures for data integration and interoperability with external sources. Double entry was conducted by a different data collector on 7% of the sample for data verification, with 98.1% of entries in agreement. Ethical approval was granted by the UCSF Human Research Protection Program, Institutional Review Board. All databases were managed in a university-managed, firewall-protected secure web-based environment. In all datasets, immigration status was masked.

### Immigrant status identification

The primary exposure of interest was immigration status, which we coded into three groups: [1] refugees and asylees who received primary clinical care at the Family Health Center, [2] non-refugee or asylee immigrant patients, and [3] US-born patients. Since EMR data does not capture social determinant of health consistently, we used several ways to identify immigration status. Refugee and asylee status was identified through NHP data from RHEIS. The NHP collects data on each refugee or asylee receiving their first comprehensive health screen, which includes mandatory screening for communicable diseases. We linked these data to EMR data using medical record numbers. Refugees and asylees were combined in the same group due to sample size, as San Francisco has a larger population of asylees, and their receipt of NHP services which offers a unique opportunity for patients to receive in-depth health education and connection to resources. The 9 patients who were identified to be former refugees or asylees, either through the presence of an NHP medical examination before 2014 or mention of refugee or asylee status in the social history in the EMR, were included in the refugee/asylee group. Non-refugee immigrant status was identified by preferred language listed other than English, need for an interpreter, and country of origin as described in the EMR. US-born status was identified if the patient did not meet the criteria for refugee, asylee, or immigrant, and if English was the preferred language, a US region was listed as a place of origin in the social history or in unstructured fields such as history of present illness, or no world region or language other than English was listed in the EMR. Here, the status for US-born patients is subject to misclassification, as this status was given in the absence of data on place of birth. However, the Family Health Center serves a high number of immigrant patients, with clinicians routinely documenting place of birth and immigration status given the community-based resources available for immigrant patients.

### Outcomes

The outcomes of interest were presence of ICD codes for non-communicable chronic diseases (hypertension, type 2 diabetes, type 2 diabetes complications [retinopathy, nephropathy, neuropathy], chronic kidney disease, coronary artery disease, hyperlipidemia, obesity, and chronic pain), chronic communicable diseases (latent tuberculosis, hepatitis B, and hepatitis C), mental health disorders (depression, anxiety, and post-traumatic stress disorder [PTSD]), and health behaviors (cigarette smoking and alcohol use disorder). These outcomes were selected as they are common health conditions diagnosed or managed in primary care settings. Data were missing on outcomes for fewer than 10 patients in each group due to clinical screening not being completed. Patients with missing data were omitted from analyses.

### Covariates

We included multiple covariates. Age was categorized (18–34 years, 35–64 years, 65 years and older). Gender was defined as woman or man, based on the self-reported gender that patients preferred. There were less than 5 patients who were identified in the EMR as transgender, and none were listed as being non-binary; they were included in the category of the gender they identified by. We included race and ethnicity (Asian and Pacific Islander, Black, Latine, Middle Eastern or North African, White, Other/unknown), which was self-identified by patients in most cases. This variable was included to capture differences in social exposures, such as racism. Here, race and ethnicity are conceptualized as social constructs. Patients who were not identified as Latine were considered non-Hispanic. While we made efforts to identify patients who were Middle Eastern or North African, it may be that some patients identified as White. We included preferred language (Arabic, Cantonese, English, Spanish, Other); and region of origin (China, Europe, Mexico, other Asia and South Pacific, other Central and South America, Middle East and North Africa, Sub-Saharan Africa, the US and Canada, and unknown). Preferred language was collapsed into a categorical variable for analyses, preferentially capturing languages other than English (i.e., if a patient listed English and Spanish as preferred languages, Spanish language was captured with this variable). We also obtained housing status (living with family, living with friend/partner, living alone, experiencing houselessness, and other/unknown), as prior work has shown that experiencing houselessness is associated with worse health outcomes across medical conditions [29]. Years living in the US was obtained for refugees/asylees and immigrants when present in the EMR. However, given that it was available for only 40 immigrant patients, it was not included in regression models. Years receiving care at the clinic was also included, based on date of first visit or laboratory result in the EMR, as more time in care may increase the likelihood of being diagnosed with non-communicable chronic diseases such as hypertension.

### Data analysis

First, data were tabulated, and chi-square statistics were conducted to identify prevalence of medical conditions by immigration status. Next, bivariate Poisson regression models were conducted to identify associations between sociodemographic characteristics and medical conditions. Lastly, multivariable Poisson regression models with robust variance assessed the association of the most common medical conditions in our sample with immigration status adjusted for age, gender, race and ethnicity, preferred language, and years receiving care at the clinic. Covariates were not included if they contained small cell sizes (i.e., housing), were highly collinear with other covariates (i.e., language and region of origin), or were missing for a substantial number of patients (i.e., years living in the US). Health outcomes that were uncommon (i.e., health behaviors) were not included in regressions. Data cleaning and analyses were conducted using Stata MP version 16.

## Results

### Patient demographic characteristics

**Fig. 1:**
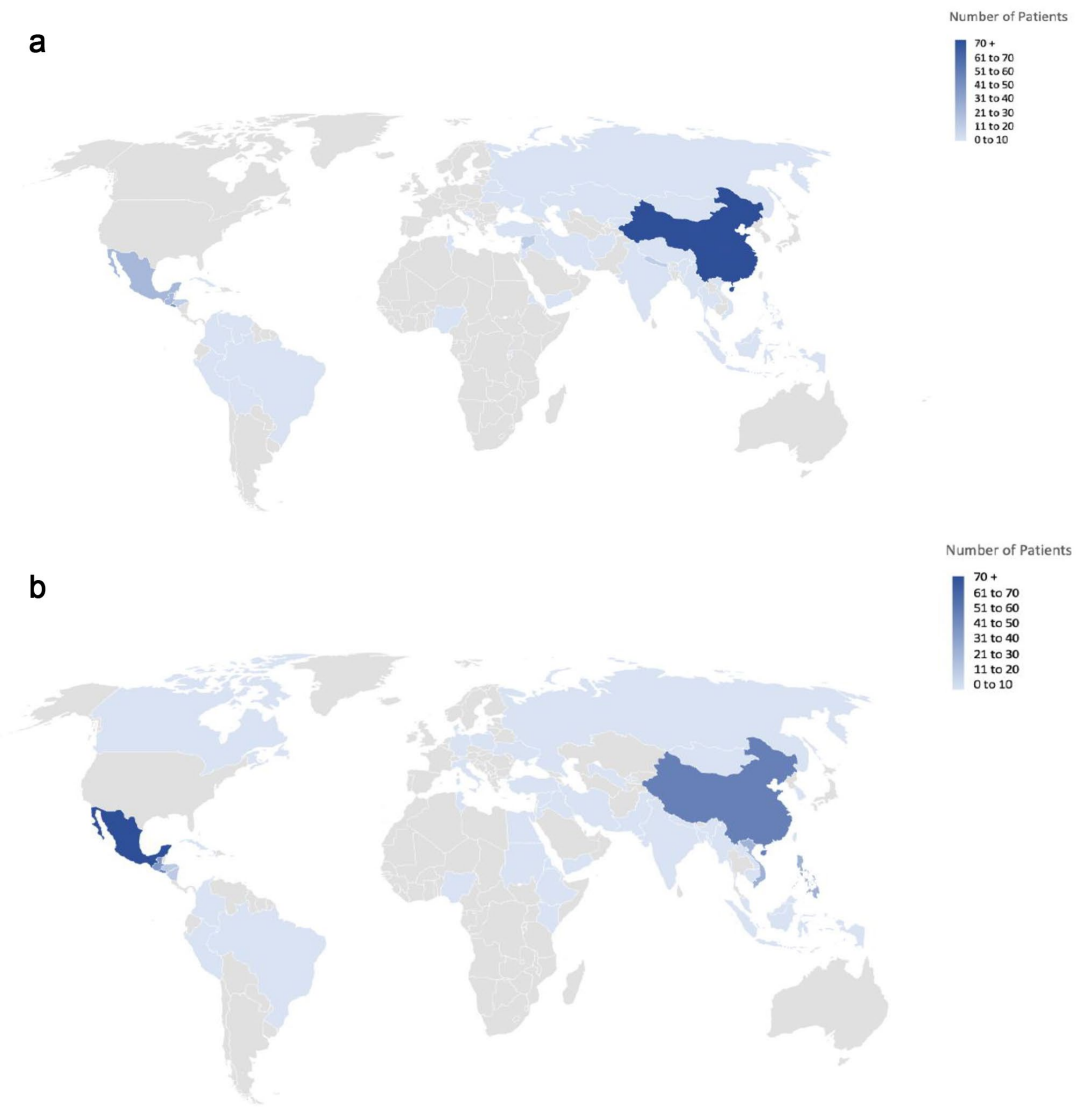
Distribution of country of origin for refugee/asylee and immigrant patients, 2014–2017 **a**. Refugee/asylee patients; Notes: Data from electronic medical records from a public safety-net clinic, 2014–2017, sample size 343. **b**. Immigrant patients; Notes: Data from electronic medical records from a public safety-net clinic, 2014–2017, sample size 450

We examined records of 343 refugees/asylee, 450 immigrant, and 202 US-born patients seen at the Family Health Center (Table 1). Women made up more than half of the sample across immigration status and 58.1% in the sample overall. The mean age for refuge/asylee patients was 35.6 years (standard deviation [SD] 12.9), immigrant patients 47.2 years (SD 16.4), and US-born patients 40.6 years (SD 16.8). Most foreign-born patients identified as Latine or Asian/Pacific Islander (refugee/asylees: 40.0% and 42.9%, immigrants: 56.0% and 26.7%, respectively). Many refugees/asylees spoke languages other than English (47.8%); a large proportion of refugee/asylee (39.7%) and immigrant patients (56.7%) listed Spanish as a preferred language. Foreign-born patients reported Central and South America, China, and Asia and the South Pacific as the most common regions of origin (Fig. 1). 30% of US-born patients were White, and less than 5% spoke languages other than English. More than half (55.7%) of patients reported living with family, and this varied across immigration status although a substantial proportion had other/unknown housing status. Refugees/asylees had been in the US for 3.4 years (SD 11.8) on average, compared to 14.4 years (SD 3.2) for immigrants. Refugee/asylees had received services at the Family Health Center for 1.7 years (SD 0.8) on average, compared to 3.4 years (SD 1.0) and 3.1 years (SD 1.0) for immigrant and US-born patients, respectively.

**Table 1:** Patient demographic characteristics by immigration status, 2014-2017

	n (%) or mean (SD)			
	US-Born	Immigrant	Refugee/Asylee	Total
	N = 202	N = 450	N = 343	N = 995
Women	114 (56.4)	286 (63.6)	178 (51.9)	578 (58.1)
Age (years)				
18–34	91 (45.0)	113 (25.1)	178 (51.9)	382 (38.4)
35–64	92 (45.5)	260 (57.8)	154 (44.9)	506 (50.9)
65 and older	19 (9.4)	77 (17.1)	11 (3.2)	107 (10.8)
Race and Ethnicity				
Asian and Pacific Islander	12 (0.7)	120 (26.7)	147 (42.9)	281 (28.2)
Black	15 (7.4)	10 (2.2)	8 (2.3)	33 (3.3)
Latine	45 (22.3)	252 (56.0)	136 (40.0)	433 (43.5)
Middle Eastern/Northern African	< 5 (< 2.5)	11 (2.4)	19 (5.5)	31 (3.1)
White	73 (36.1)	10 (2.2)	6 (1.7)	89 (8.9)
Other/Unknown	54 (26.7)	47 (10.4)	27 (7.9)	128 (12.9)
Language^a^				
Arabic	< 5 (< 2.5)^b^	14 (3.1)	30 (8.7)	45 (4.5)
Cantonese	0 (0)	47 (10.4)	12 (3.5)	59 (5.9)
English	197 (97.5)	112 (24.9)	61 (17.8)	370 (37.2)
Spanish	5 (2.5)	255 (56.7)	136 (39.7)	396 (39.8)
Other	< 5 (< 2.5)^b^	81 (18.0)	164 (47.8)	246 (24.7)
Region of Origin				
China		43 (9.6)	77 (22.4)	120 (12.1)
Europe		15 (3.3)	20 (5.8)	35 (3.5)
Mexico		69 (15.3)	24 (7.0)	93 (9.3)
Middle East/Northern Africa		22 (4.9)	44 (12.8)	66 (6.6)
Other Central and South America		116 (25.8)	106 (30.9)	222 (22.3)
Other Asia and South Pacific		82 (18.2)	49 (14.3)	131 (13.2)
Sub-Saharan Africa		9 (2.0)	10 (2.9)	19 (1.9)
US and Canada	202 (100)	13 (2.9)	8 (2.3)	223 (22.4)
Unknown		81 (18.0)	5 (1.5)	86 (8.6)
Housing				
Family	72 (35.6)	288 (64.0)	194 (56.6)	554 (55.7)
Friend/Partner	25 (12.4)	38 (8.4)	54 (15.7)	117 (11.8)
Alone	10 (5.0)	26 (5.8)	37 (10.8)	73 (7.3)
Homeless	12 (5.9)	5 (1.1)	< 5 (< 1.5)^b^	21 (2.1)
Other/Unknown	83 (41.0)	93 (20.7)	54 (15.7)	230 (23.1)
Years in the U.S.		14.4 (11.8)	3.4 (3.2)	7.8 (9.6)
Years receiving care at the clinic	3.1 (1.0)	3.4 (1.0)	1.7 (0.8)	2.8 (1.2)

Notes: Data from electronic medical records from a public safety-net clinic, 2014–2017, SD = standard deviation

^a^ Not mutually exclusive here, but collapsed into a categorical variable for analyses

^b^ Percentages not calculated for small cell sizes, instead reported as < 5/n*100 (i.e., < 5/202 = < 2.5)

### Prevalence of medical conditions by immigration status

We first examined unadjusted prevalence by chi-square of each condition by immigration status. Overall, non-communicable chronic diseases were common amongst all groups (Table 2), but less so in refugees/asylees. About 8% of refugees/asylees were diagnosed with hypertension, compared with 32.1% of immigrants and 30.2% of US-born patients. Type 2 diabetes was reported in 5.2% refugees/asylees, compared with 21.7% of immigrants and 11.9% of US-born patients. Hyperlipidemia was diagnosed in 7% of refugees/asylees, 23.9% immigrants, and 12.4% of US-born patients. About 10% of refugees/asylees were obese, whereas almost 19.3% of immigrants and 17.9% of US-born patients were obese. Chronic pain was identified in 19.5% of refugees/asylees, compared with 37.8% of immigrants and 29.7% of US-born patients.

**Table 2:** Prevalence of medical conditions by immigration status, 2014–2017

	n (%)				p-value
	US-Born	Immigrant	Refugee/Asylee	Total	
	N = 202	N = 450	N = 343	N = 995	
Chronic non-communicable diseases					
Hypertension	61 (30.2)	144 (32.1)	28 (8.2)	233 (23.5)	< 0.001
Type 2 diabetes	24 (11.9)	97 (21.7)	18 (5.2)	141 (14.1)	< 0.001
Type 2 diabetes-related comorbidities	10 (5.0)	32 (7.2)	5 (1.5)	47 (4.7)	< 0.001
Chronic kidney disease	13 (6.4)	18 (4.0)	< 5 (< 1.5)^a^	33 (3.3)	< 0.001
Coronary artery disease	9 (4.5)	17 (3.8)	< 5 (< 1.5)^a^	28 (2.8)	0.04
Hyperlipidemia	25 (12.4)	107 (23.9)	24 (7.0)	156 (15.6)	< 0.001
Obesity	36 (17.9)	86 (19.3)	34 (10.1)	156 (15.6)	0.001
Chronic pain	60 (29.7)	169 (37.8)	67 (19.5)	294 (29.4)	< 0.001
Chronic communicable diseases					
Latent tuberculosis	< 5 (< 2.5)^a^	38 (8.5)	17 (5.1)	59 (5.9)	0.003
Hepatitis B	< 5 (< 2.5)^a^	9 (0.9)	13 (2.9)	24 (2.4)	0.04
Hepatitis C	< 5 (< 2.5)^a^	6 (1.3)	< 5 (< 1.5)^a^	12 (1.2)	0.33
Mental illnesses					
Depression	54 (26.7)	99 (22.1)	63 (18.4)	218 (21.8)	0.07
Anxiety	34 (16.8)	57 (12.8)	42 (12.2)	132 (13.2)	0.13
Post-traumatic stress disorder	5 (2.5)	16 (3.6)	47 (13.7)	68 (6.8)	< 0.001
Health behaviors					
Cigarette smoking	32 (15.8)	33 (7.3)	32 (9.3)	97 (9.8)	0.003
Alcohol use disorder	19 (9.4)	24 (5.3)	15 (4.3)	58 (5.8)	0.04

Note: Data from names of diagnoses listed in electronic medical records from a public safety-net clinic, 2014–2017. P-values listed from chi-square testing

^a^ Percentages not calculated for small cell sizes, instead reported as < 5/n*100 (i.e., < 5/202 = < 2.5)

The prevalence of chronic communicable diseases also differed by immigration status. Latent tuberculosis was identified in 5.1% of refugees/asylees, 8.5% of immigrants, and < 2.5% of US-born patients. Hepatitis B and C were identified in 2.9% and < 1.5% of refugees/asylees, 0.9% and 1.3% immigrants, and < 2.5% and < 2.5% of US-born patients, respectively. Since there were only 10 patients diagnosed with HIV in our sample, we did not include this diagnosis in the present study.

Overall, the prevalence of mental illnesses in all patient groups was high, although different groups were at higher risk for different conditions. Depression and anxiety were diagnosed in 18.4% and 12.2% of refugees/asylees, in 22.1% and 12.8% of immigrants, and in 26.7% and 16.8% of US-born patients, respectively. PTSD was diagnosed in 13.7% of refugees/asylees, 3.6% of immigrants, and 2.5% of US-born patients.

Risky health behaviors were prevalent. Amongst refugees/asylees, 9.3% smoked cigarettes and 4.3% were identified to have alcohol use disorder. Amongst immigrants, 7.3% smoked cigarettes and 5.3% had alcohol use disorder. Amongst US-born patients, 15.8% smoked cigarettes and 9.4% had alcohol use disorder.

### Association between patient sociodemographic characteristics and medical conditions

In bivariate Poisson regression models (Supplemental Table 2), compared to refugee/asylee patients (reference group), US-born and immigrant patients were more likely to be diagnosed with hypertension (US-born: estimated relative risk [IRR] [CI (95% confidence interval)] = 3.5 [2.4,5.6]; immigrant: IRR[CI] = 3.9 [2.7,5.7]), type 2 diabetes (US-born: IRR [CI] = 2.3 [1.2,4.0]; immigrant: IRR[CI] = 4.1 [2.5,6.7]), hyperlipidemia (US-born: IRR [CI] = 1.7 [1.0,3.0]; immigrant: IRR[CI] = 3.4 [2.2,5.2]), and chronic pain (US-born: IRR [CI] = 1.5 [1.1,2.0]; immigrant: IRR[CI] = 1.9 [1.5,2.5]). US-born patients were more likely to be diagnosed with depression (IRR [CI] = 1.5 [1.1,2.0]). Both US-born.

and immigrant patients were less likely to be diagnosed with PTSD (US-born: IRR [CI] = 0.2 [0.1,0.4]; immigrant: IRR[CI] = 0.2 [0.1,0.4]).

In multivariable Poisson regression models with robust variance (Table 3), compared with refugees/asylees (reference group), US-born patients were more likely to be diagnosed with hypertension (IRR [CI] = 1.8 [1.0, 3.7]) and less likely to be diagnosed with depression (IRR[CI] = 0.5 [0.3, 0.8]). Both US-born and immigrant patients were less likely to be diagnosed with PTSD (US-born patients: IRR[CI] = 0.06 [0.01, 0.2], immigrant patients: IRR[CI] = 0.1 [0.06, 0.2]), compared with refugee/asylee patients. Sensitivity analyses showed that the reversal in the association between depression and immigration status was caused by the preferred language variable, which might be responsible for some confounding.

**Table 3:** Association between patient group and sociodemographic characters and common medical conditions, multivariable analyses

Demographic characteristics	IRR (95% CI; p-value)							
	Hypertension	Type 2 diabetes	Hyperlipidemia	Chronic pain	Latent tuberculosis	Depression	Anxiety	Post-traumatic stress disorder
Immigration status (Ref: refugee/asylee)								
US-born	1.8 (1.0,3.7; 0.04) *	1.6 (0.7,3.7; 0.3)	1.2 (0.5,2.8; 0.6)	0.9 (0.5,1.5; 0.7)	0.8 (0.2,4.1; 0.8)	0.5 (0.3,0.8; 0.006) **	1.1 (0.4,2.9; 0.9)	0.06 (0.01,0.2; 0.00) **
Immigrant	1.5 (1.0,2.3; 0.07)	1.2 (0.7,2.2; 0.5)	1.4 (0.8,2.4; 0.2)	1.0 (0.7,1.4; 1.0)	1.5 (0.8,3.0; 0.2)	0.7 (0.5,1.1; 0.1)	0.7 (0.4,1.2; 0.4)	0.1 (0.06,0.2; 0.00) **
Age (Ref: 18–34 years)								
35–64 years	5.7 (3.3,9.8; 0.00) **	9.5 (4.1,21.7; 0.00) **	7.1 (3.4,14.8; 0.00) **	1.4 (1.1,1.8; 0.003) **	0.8 (0.5,1.5; 0.6)	1.1 (0.8,1.4; 0.7)	1.2 (0.8,1.7; 0.3)	1.5 (0.9,2.5; 0.08)
>65 years	12.7 (7.4,22, 0.00) **	16.2 (6.9,38.0; 0.00) **	13.9 (6.4,30.0; 0.00) **	1.1 (0.8,1.6; 0.5)	0.4 (0.1.1.3; 0.1)	1.1 (0.7,1.7; 0.6)	0.9 (0.5,1.7; 0.7)	1.0 (0.3.3.4; 1.0)
Gender (Ref: men)								
Women	0.7 (0.6,0.8; 0.00) **	1.1 (0.8,1.5; 0.5)	0.5 (0.4,0.7; 0.00) **	1.0 (0.8,1.2; 0.8)	1.1 (0.6,1.8; 0.8)	1.7 (1.3,2.2; 0.00) **	1.6 (1.1,2.2; 0.008) **	1.7 (1.1,2.7; 0.02) *
Race and Ethnicity (Ref: White)								
Asian & Pacific Islander	1.59 (1.0,2.6; 0.06)	3.8 (1.2,11.7; 0.02)*	1.8 (0.8,3.9;0.1)	0.9 (0.6,1.5; 0.8)	1.1 (0.3,4.7; 0.9)	0.4 (0.2,0.6; 0.00) **	0.4 (0.2,1.1; 0.07)	0.2 (0.08,0.7; 0.009)**
Black	1.3 (0.8,2.2; 0.3)	1.4 (0.4,5.3; 0.6)	0.8 (0.2,2.4;0.7)	1.7 (1.1,2.7; 0.02) *	0.9 (0.1,7.8; 0.9)	0.6 (0.3,1.2; 0.1)	1.0 (0.4,2.2; 0.9)	0.5 (0.08,2.8; 0.4)
Latine	1.7 (1.1,2.7; 0.03) *	3.0 (1.1,8.1; 0.03) *	1.5 (0.7,3.3; 0.3)	0.9 (0.6,1.6; 0.8)	0.8 (0.1,5.2; 0.8)	0.6 (0.4,1.0; 0.04) *	0.7 (0.3,1.6; 0.5)	0.9 (0.3, 2.7; 0.9)
Middle Eastern/North African	0.7 (0.2,2.2; 0.6)	2.6 (0.4,17.6; 0.3)	0.4 (0.0,4.0;0.4)	1.9 (0.9,3.7; 0.08)	1.0 (0.2,6.3; 1.0)	1.0 (0.4,2.1; 1.0)	1.2(0.4,3.8; 0.7)	0.9 (0.09,8.6; 0.9)
Other	1.3. (0.9,2.2)	2.6 (0.97,7.2; 0.06)	1.7 (0.9,3.3; 0.1)	1.0 (0.6,1.5; 0.9)	0.3 (0.0,2.2; 0.2)	0.5 (0.3,0.8; 0.009)**	0.3 (0.2,0.8; 0.01)*	0.6 (0.2,1.9; 0.4
Language (Ref: English)								
Arabic	0.3 (0.0,1.3; 0.09)	1.1e-06 (4.1e-07,3.1e-06;0.00)**	0.9 (0.2,3.9;0.9)	0.7 (0.4,1.5; 0.4)	0.9 (0.2,4.3; 0.9)	0.6 (0.3,1.4; 0.2)	1.1 (0.3,3.2; 0.9)	1.1 (0.1,9.1;0.9)
Cantonese	0.7 (0.4,1.0; 0.07)	0.7 (0.3,1.3; 0.2)	0.9 (0.5,1.8;0.9)	0.9 (0.5,1.6; 0.7)	2.8 (0.7,11.1; 0.1)	0.3 (0.1,1.0; 0.04) *	0.7 (0.2,2.8; 0.6)	0.5 (0.06, 4.3; 0.5)
Spanish	0.7 (0.4,1.0; 0.07)	1.3 (0.6,2.8; 0.5)	1.0 (0.5,2.1;0.9)	1.2 (0.7,1.9; 0.5)	2.5 (0.4,15.4; 0.3)	0.6 (0.4,1.0; 0.7)	1.2 (0.5,3.3; 0.7)	0.5 (0.1,1.9; 0.3)
Other	0.9 (0.6,1.2; 0.5)	0.6 (0.3,1.0; 0.07)	1.0 (0.5,1.7;0.9)	0.9 (0.6,1.4; 0.8)	2.1 (0.6,7.4; 0.2)	0.4 (0.2,0.6; 0.00) **	0.9 (0.4,2.1; 0.8)	0.5 (0.2,1.6; 0.2) *
Years at the clinic	1.3 (1.2,1.5; 0.00) **	1.3 (1.1,1.5; 0.001) **	1.3 (1.1,1.5; 0.00) **	1.4 (1.3,1.5; 0.00) **	1.2 (0.9,1.5; 0.2)	1.1 (1.0,1.3; 0.03)*	1.1 (0.9,1.3; 0.2)	1.3 (1.0,1.6; 0.04)*

Note: Poisson regression model with robust variance. Data from electronic medical records from a public safety-net clinic, 2014–2017. IRR = estimated relative risk, CI: confidence interval. Based on a sample of 343 refugees/asylee, 450 immigrant, and 202 US-born patients. * p < 0.05, ** p < 0.01

## Discussion

This study fills a critical gap in the literature by comparing disease burden in refugee/asylee, non-refugee immigrant, and US-born patients at the largest safety net primary care clinic in San Francisco. We found that differences in disease burden by immigration status in adjusted analyses were partially explained by differences in sociodemographic characteristics. In adjusted models, refugees/asylees were more likely to be diagnosed with a mental health disorder and less likely to be diagnosed with hypertension compared with US-born patients.

While non-communicable chronic diseases were common across immigration status, they were less prevalent among refugees/asylees in both unadjusted and adjusted analyses. Our findings vary from prior reports. Berkowitz et al. included refugees from Somalia, Bhutan, and Iran, amongst other countries, compared them to Spanish-speaking immigrants and US-born patients and followed patients over a period of 10 years; they found that refugees and immigrants had a greater risk of being diagnosed with non-communicable chronic diseases compared to US-born patients [22]. Reed et al., using the National Immigrant Survey, found that refugees had greater odds of reporting chronic medical conditions and poor health compared to non-refugee immigrants [30]. Norredam et al. followed refugees and immigrants reunited with family compared with Danish-born patients over 20 years and found that refugees and immigrants had lower initial risk for cardiovascular outcomes within 5 years of arrival compared with Danish-born patients, however, after longer follow-up, that risk became higher than that of Danish-born patients [31]. The differences in disease burden in our patient population may be partially explained by differences in age, in that refugees/asylees were on average younger. Alternatively, immigrant and US-born patients had longer follow-up time overall and may have been more likely to receive a chronic disease diagnosis compared with refugees/asylees. In our sample, refugees and asylees came from the same world regions as immigrants. It may be that the refugees and asylees represent a younger sample of the same population of immigrants in our study. In addition, the services and resources available to refugees and asylees could have had a protective effect compared to non-refugee immigrants.

The findings from our study, that there were few differences between refugees/asylees and US-born patients for most medical conditions, except for hypertension, are not consistent with prior work highlighting a “healthy migrant effect,” where it is postulated that those arriving in a new country are healthier at baseline than others who stay behind and subsequently healthier than US-born individuals in the host country [32]. This paradox has been contradicted and is still under scrutiny as further analyses have identified other patterns in health burden [33]. Our findings also do not corroborate the “refugee health disadvantage,” whereby refugees have poorer health outcomes overall compared to non-refugee immigrants [30].

When compared to refugees/asylees in multivariable analyses, immigrants and US-born patients were less likely to have a mental health diagnosis. While unadjusted analyses found that US-born patients were more likely to be diagnosed with depression compared to refugee/asylee patients, this association was reversed in multivariable models due to confounding caused by preferred language. Refugee and asylees are often exposed to various physical and emotional trauma before and during their migration as well as stressors during resettlement and integration, which may further worsen mental health [34, 35]. The lifetime prevalence among the general US population is 6.8% for PTSD and 12% for any depressive disorder [36, 37]. Similar to our findings, a recent study noted that refugees experience a high rate of mental health disorders, particularly depression and PTSD [7]. Here, the “refugee health disadvantage” may be at play, where the health of this population is negatively impacted, possibly stemming from trauma and exposure to violence [30]. Nevertheless, it may also be that there are fundamental differences in screening practices for PTSD in the broader clinic patient population. Patients receiving care at the Family Health Center come from communities that are historically disadvantaged, and as a result, have low socioeconomic status. The association of low socioeconomic status with adverse mental health outcomes has been well established [38]. Indeed, US-born patients in our study had more than twice the national prevalence of depression. However, PTSD prevalence was lower than expected. Refugees and asylees are compulsorily screened for PTSD as part of the Refugee Health Assessments. Primary care providers may be underscreening other patients for PTSD, which could potentially drive this stark difference.

The differences between this study and prior work may also be because refugee and asylee populations resettled in the San Francisco Bay Area have different health profiles compared to refugees in other parts of the country. In fact, most patients who received services through the NHP had received asylee status, rather than refugee status. Refugee populations not only receive support from the Resettlement Agency but, given their arrival in clusters as a result of urgent human rights crises, also benefit from focused responses from community- and faith-based organizations, foundations, and businesses alike [17, 39]. Asylee populations usually arrive alone or in smaller family units, apart from the more recent migrant “caravans” which have been widely reported on, and while resources exist to support new asylees, they are fewer [39]. Moreover, due to the legal implications of applying for asylum—refugees are granted status in their home countries while asylees request status in the host country—and the nature of the reasons for seeking asylum, asylees may experience more personalized persecution. The differences in disease burden we found between refugees/asylees and immigrants may be in part explained by differential access to resources, socioeconomic factors, or effects from experiences of persecution.

There have been recent efforts to capture social determinants of health in clinical settings [40]. Connecting immigrant patients, including those who hold refugee or asylee status, to community-based organizations is one of multiple reasons to capture immigration status in EMR. Another consideration is the optimization of referrals to such resources. For example, a machine learning algorithm, based on geographic location and refugee characteristics, was developed to improve financial outcomes for refugees during the resettlement period [41]. Researchers have found that this algorithm has increased refugees’ probability of securing employment after 90 days by up to 50%. The ethical dilemma in documenting immigration status in EMR should be weighed, however, given the hypothetical risk of capturing sensitive legal information even in a system which is meant to store protected information [42].

This study has several strengths. We assess differences in multiple health conditions in a large population of refugee/asylee, immigrant, and US-born patients in a major urban public safety net clinic over 3 years. There are also several limitations. First, this study is based on manual chart review and some sociodemographic characteristics may not have been well documented by providers. Second, presence of medical conditions was ascertained from diagnoses listed by primary care providers. Some conditions may have been missed if patients were not screened. Third, if a patient listed English as their preferred language and there was no mention of a country of birth, patients were assumed to have been born in the US, which may have led to misclassification. However, the clinic serves a large proportion of immigrants, and clinicians typically record robust social histories, listing at least country of origin. Future work should include consistent recording of patient demographic characteristics and social determinants of health in clinical records given their importance in health outcomes. Fourth, unlike most resettlement agencies, the Newcomers Health Program is located within the clinic and serves as a continued resource for refugees and asylees who receive their care in the clinic; this may limit the generalizability of our findings. Fifth, we did not adjust p-values for multiple hypothesis testing. It may be that the association between diagnosis with hypertension and immigration status would not hold with such adjustment given that the associated p-value is close to 0.05. Nevertheless, associations between mental health diagnoses and immigration status would most likely still hold after adjustment. Lastly, our data included patients seen from 2014 to 2017. While there have been more refugee groups welcomed in California since, most have resettled outside of the San Francisco Bay Area due to high cost of living. Moreover, as a ramification of stringent immigration policies from the Trump administration, more recent numbers of people seeking asylum and receiving asylee status in San Francisco have been lower than during prior years [43].

## Conclusions

Here, we found important differences in disease burden between refugee/asylee, immigrant, and US-born patients. There are several public health implications that stem from this study. First, our findings have important implications for clinical screening practices, particularly in clinics that serve refugees and immigrants without the dedicated resources provided by clinics like the Newcomer Health Program described here. Second, this report provides valuable data to guide resource allocations. Clinical approaches seeking to improve the lives of forcibly displaced people benefit from robust data on longer-term health outcomes, beyond the first few months in a welcoming country, and this study contributes to this literature.

## Electronic supplementary material

Below is the link to the electronic supplementary material.

Supplementary Material 1

Supplementary Material 2

## Data Availability

The datasets generated and analysed during the current study are not publicly available due the sensitive nature of the data (immigration status) but are available from the corresponding author on reasonable request.

## List of Abbreviations

CI: Confidence intervals
EMR: Electronic medical records
IRR: Estimated relative risk
PTSD: Post-traumatic stress disorder
SD: Standard deviation
US: United States
