# Supplementary material for: Comparing the health of refugee and asylee patients with that of non-refugee immigrant and US-born patients in a large Urban clinic
Source: BMC Public Health. 2023 Jul 27;23:1438. doi: 10.1186/s12889-023-16349-5 (PMC10373359; doi:10.1186/s12889-023-16349-5)
Supplement: Supplementary file 1 — Supplementary Material 1 [file 12889_2023_16349_MOESM1_ESM.docx]

Supplemental Table 1. Data sources and data collected

| Immigration status group | Data sources | Data collected |
| --- | --- | --- |
| All patients | Electronic medical records | Demographic characteristics as documented by primary care physician, past medical history, past surgical history, medical conditions (ICD codes), prescriptions |
| Refugees and asylees | Refugee health screening data (entered in electronic medical records) | Place of birth, years living in country of birth, educational attainment, past and current occupation, reason for leaving home country, type of persecution experienced, screening for PTSD |
