# Supplementary material for: Comparing the health of refugee and asylee patients with that of non-refugee immigrant and US-born patients in a large Urban clinic
Source: BMC Public Health. 2023 Jul 27;23:1438. doi: 10.1186/s12889-023-16349-5 (PMC10373359; doi:10.1186/s12889-023-16349-5)
Supplement: Supplementary file 2 — Supplementary Material 2 [file 12889_2023_16349_MOESM2_ESM.docx]

Supplemental table 2. Association between patient group and sociodemographic characters and common medical conditions, bivariate analyses

|  | IRR (95% CI) | | | | | | | | |
| --- | --- | --- | --- | --- | --- | --- | --- | --- | --- |
|  | HTN | Type 2 Diabetes | HLD | Chronic Pain | LTBI | Depression | Anxiety | PTSD |  |
| Group (Ref: refugee patients) |  |  |  |  |  |  |  |  |  |
| US-born patients | 3.7 (2.4,5.6) ** | 2.3 (1.2,4.0) ** | 1.7 (1.0,3.0) * | 1.5 (1.1,2.0) ** | 0.4 (0.1,1.1) | 1.5 (1.1,2.0) * | 1.3 (0.9,2.0) | 0.2 (0.1,0.4) ** |  |
| Immigrant patients | 3.9 (2.7,5.7) ** | 4.1 (2.5,6.7) ** | 3.4 (2.2,5.2) ** | 1.9 (1.5,2.5) ** | 1.7 (1.0,3.0) | 1.2 (0.9,1.6) | 1.0 (0.7, 1.5) | 0.2 (0.1,0.4) ** |  |
| Age (Ref: 18-34 years) |  |  |  |  |  |  |  |  |  |
| 35-64 years | 7.5 (4.4,12.7) ** | 11.7 (5.2,26.5) ** | 9.4 (4.6,19.2) ** | 18 (1.4,2.3) ** | 1.2 (0.7,2.1) | 1.0 (0.7,1.3) | 1.2 (0.8,1.6) | 1.0 (0.6,1.7) |  |
| >65 years | 20.4 (12,34.5) ** | 24.6 (10.8,56.5) ** | 21.6 (10.6, 44.3) ** | 1.7 (1.3, 2.4) ** | 0.7 (0.2,2.0) | 1.0 (0.7,1.6) | 0.8 (0.4,1.5) | 0.4 (0.1,1.3) |  |
| Gender (Ref: Male) |  |  |  |  |  |  |  |  |  |
| Female | 0.8 (0.7,1.1) | 1.4 (1.0,2.0) * | 0.7 (0.5,0.9) * | 1.1 (0.9,1.3) | 1.2 (0.7,2.0) | 1.5 (1.2,2.0) ** | 1.5 (1.0,2.1) * | 1.5 (0.9,2.4) |  |
| Race/ethnicity (Ref: White) |  |  |  |  |  |  |  |  |  |
| Asian & Pacific Islander | 1.1 (0.7,1.6) | 2.3 (0.9,5.8) | 1.6 (0.9,3.0) | 0.9 (0.6,1.3) | 2.2 (0.7,7.3) | 0.3 (0.2,0.5) ** | 0.4 (0.2,0.7) ** | 0.7 (0.2,2.3) |  |
| Black | 1.7 (0.9,3.0) | 1.9 (0.5,7.5) | 0.9 (0.3,3.2) | 2.1 (1.3,3.3) ** | 1.0 (0.1,9.8) | 0.9 (0.5,1.7) | 1.1 (0.5,2.4) | 0.8 (0.1,6.8) |  |
| Latinx | 1.0 (0.6,1.5) | 3.3 (1.4,7.9) ** | 1.5 (0.8,2.8) | 1.2 (0.8,1.8) | 2.1 (0.7,6.8) | 0.8 (0.6,1.1) | 0.8 (0.5,1.3) | 2.1 (0.8,5.6) |  |
| Middle Eastern/North African | 0.3 (0.1,1.1) | 0.6 (0.1,4.7) | 0.3 (0.0,2.1) | 1.2 (0.6,2.2) | 1.0 (0.1,8.9) | 1.1 (0.6,1.9) | 1.0 (0.4,2.2) | 4.3 (1.3,14.3)* |  |
| Other | 0.9 (0.5,1.5) | 1.7 (0.6,4.7) | 1.3 (0.6,2.6) | 0.9 (0.6,1.4) | 0.6 (0.1,2.6) | 0.7 (0.4,1.0)** | 0.3 (0.2,0.7) * | 1.3 (0.4,4.3) |  |
| Housing (Ref: Housed) |  |  |  |  |  |  |  |  |  |
| Unhoused | 1.3 (0.7,2.7) | 1.4 (0.6,3.5) | 1.6 (0.7,3.4) | 1.7 (1.2,2.7) * | - | 1.3 (0.6,2.6) | 1.0 (0.3,3.0) | 2.7 (1.1,7.0) * |  |
| Language (Ref: English) |  |  |  |  |  |  |  |  |  |
| Spanish | 0.7 (0.5,0.8) ** | 1.4 (0.9,2.0) | 1.2 (0.8,1.7) | 1.1 (0.9,1.4) | 3.3 (1.4,7.9) ** | 0.9 (0.7,1.1) | 1.1 (0.7,1.6) | 3.2 (1.5,6.7) ** |  |
| Cantonese | 1.1 (0.7,1.7) | 1.5 (0.8,2.8) | 2.1 (1.2,3.5) ** | 0.9 (0.6,1.5) | 5.1 (1.7,15.3) ** | 0.3 (0.1,0.7) ** | 0.4 (0.1,1.2) | 0.6 (0.1,5.0) |  |
| Arabic | 0.1 (0.0, 0.5) ** | - | 0.3 (0.1,1.3) | 0.7 (0.4,1.2) | 1 (0.1,8.1) | 0.9 (0.5,1.5) | 0.9 (0.4,2.0) | 5.2 (2.0,13.7) ** |  |
| Other | 0.7 (0.5,0.9) * | 0.6 (0.4,1.0) | 1.0 (0.6,1.5) | 0.7 (0.6,1.0) | 2.8 (1.1,7.0) * | 0.3 (0.2,0.5) ** | 0.5 (0.3,0.9) * | 1.7 (0.7,4.0) |  |
| Years at FHC | 1.7 (1.6,1.9) ** | 1.7 (1.5,1.9) ** | 1.6 (1.4,1.8) ** | 1.4 (1.3,1.5) ** | 1.2 (1.0,1.4) | 1.2 (1.0,1.3) ** | 1.1 (1.0,1.3) | 0.8 (0.7,1.0) * |  |

Note: bivariate Poisson regression models. HTN = hypertension, HLD = hyperlipidemia, FHC = Family Health Center, LTBI = latent tuberculosis, PTSD = post-traumatic stress disorder. * p < 0.05, ** p <0.01
